# Exploring the therapeutic potential of momordica balsamina: A focus on anticancer and nephroprotective effects

**DOI:** 10.1007/s11033-026-12383-9

**Published:** 2026-07-20

**Authors:** Mante Kgakishe, Marole Maluleka, Kgomotso Poopedi, Leseilane Mampuru, Vusi Mbazima

**Affiliations:** 1 Department of Biochemistry, Microbiology and Biotechnology, School of Molecular and Life Sciences, University of Limpopo, Private Bag X1106, Sovenga, 0727 South Africa; 2 Research Center for Synthesis and Catalysis, Department of Chemical Sciences, University of Johannesburg, Auckland Park, South Africa

**Keywords:** *Momordica balsamina*, PARP1/p53 signaling, Cisplatin, Nephroprotective

## Abstract

**Background:**

Despite being considered a gold standard chemotherapeutic drug, cisplatin is associated with dose-limiting toxicity, especially nephrotoxicity. This highlights the need for safe and effective adjuvant agents that protect against cisplatin-induced toxicity without affecting its anticancer activity. This study investigated the potential of *Momordica balsamina* aqueous extract (MBAE) as an adjuvant to reduce cisplatin-induced kidney cell damage and death.

**Methods:**

The phytochemical analysis of the extract was done using Gas Chromatography-Mass Spectrometry. The effects of the extract in combination with cisplatin on cell viability in 2D and 3D cultures of HEK-293 and MDA-MB-231 cells were assessed using CCK-8, CellTiter-Glo 3D, and spheroid growth assays, respectively. Cell death in 2D HEK-293 and MDA-MB-231 cultures was assessed using Annexin V-FITC. DNA damage and cell cycle progression were analyzed using multicolor DNA damage and cell cycle analysis assays, respectively, while protein expression was determined by Western blotting in 2D HEK-293 cells.

**Results:**

A sum of 26 compounds, ranging from terpenoids, furans, steroids, flavanols and azaflavanols were tentatively identified. The combinatorial treatments increased HEK-293 viability in 2D and 3D cultures as well as enhanced spheroid diameter and growth. Comparatively, the co-treatment increased MDA-MB-231 cell viability in 2D models following treatment with 800 µg/ml at 24 h, while significantly reducing viability at 48 h. No notable changes in MDA-MB-231 spheroid diameters were observed although growth rate and spheroid viability decreased. The combinatory treatments increased early apoptotic cell death in a time-dependent manner in HEK-293 cells. On the other hand, a concentration- and time-dependent increase in late apoptotic cells percentages was observed in MDA-MB-231 cells. Cell cycle analysis revealed MBAE modulated the cell cycle by halting cells in the G0/G1-phase following treatment with 200 µg/ml while 800 ug/ml supplementation promoted cell cycling by shifting cell cycle arrest from the S-phase to the G2/M phase. Furthermore, a concentration-dependent decrease in pATM, pH2A.X, and co-activation was observed, although DNA damage worsened after 48 h of exposure. The combinatorial treatments modulated Bax/Bcl-2 ratios while upregulating p53, p21, and PARP1 protein expression to prioritize DNA repair over cell death.

**Conclusion:**

The data indicate that the extract has significant potential as a renoprotective agent in cisplatin therapy.

**Supplementary Information:**

The online version contains supplementary material available at 10.1007/s11033-026-12383-9.

## Introduction

Cisplatin is a platinum-based tumoricidal agent that has been used in the “war against cancer” for over 4 decades [1]. The widely held view of its fundamental mechanism of action is that it retards DNA repair [2], resulting in apoptotic cell death. Cisplatin activation begins in the cytosol through hydrolysis, where it produces an electrophilic compound. This activated form is transferred to the nucleus, where it forms covalent bonds with nitrogen donor atoms in nucleic acids, creating interstrand and intrastrand crosslinks [3]. Crosslinking disrupts the unwinding of the double-stranded DNA helix, blocking DNA synthesis and replication, leading to cell cycle arrest and apoptosis [4]. Alternatively, crosslinking triggers the activation of DNA repair response (DRR) mechanisms through the ataxia-telangiectasia mutated (ATM), the ataxia-telangiectasia and Rad-3-related protein (ATR), and the PARP1/p53 pathways to support cell survival [5]. PARP-1 plays a multifactorial role in cell death vs. survival and is a reliable target for designing therapeutic approaches that limit therapy resistance. In renal proximal tubular cells, the DNA crosslinks induced by cisplatin stimulate PARP1 activation, which promotes base excision repair. When excessively activated, this process may lead to the depletion of intracellular NAD⁺ and ATP, thereby shifting the equilibrium from DNA repair mechanisms to necrotic cell death and inflammatory responses [6]. A balance in the activation of PARP1 is essential when investigating nephroprotective agents that prioritize DNA damage response (DDR) as an ameliorative mode of action.

In clinical practice, the use of cisplatin is limited by various organ toxicities, including hepatotoxicity, cardiotoxicity, ototoxicity, and nephrotoxicity [7]. Advances have been made in the development of systemic approaches to bypass cisplatin–related adverse effects. These include the synthesis of carboplatin and oxaliplatin analogs. Although these analogs are less toxic, their efficacy is incomparable to that of cisplatin [8]. Cisplatin-induced adverse effects prevail, with nephrotoxicity remaining a major limitation [9]. Thus, novel strategies, especially adjuvant therapies with medicinal plants, are at the forefront of disease alleviation due to their potential to minimize chemotherapy-related toxicity while maintaining drug efficacy [10].

Natural products and their derived biomolecules have long served as reservoirs for chemopreventive and therapeutic agents. Technological advances in analytical tools and genome mining have revitalized interest in natural product-based drug discovery, particularly for mitigating chemotherapy-induced toxicities [11]. Newman and Cragg (2020) documented that over 60% of approved anticancer drugs between 1981 and 2019 were derived from or inspired by natural products [12]. *Momordica balsamina (M. balsamina)* is a plant used in various traditional medicinal systems to treat infectious and non-communicable diseases (NCDs). It is native to many regions of Southern Africa, India, Australia, and tropical Asia [13]. Like other members of the Cucurbitaceae family, cucurbitacins are the primary phytoconstituents of this species. The leaves and stems of *M. balsamina* are the primary sources of these triterpenes. A study analyzing a leaf methanol extract of *M. balsamina* identified 10 flavanols recognized for their antioxidative effects [14]. Another investigation revealed three glycosidic cucurbitane-type triterpenoids, three flavanol glycosides, and five cucurbitane-type triterpenoids [15]. These phytoconstituents have been reported to have antimetastatic [16] and antioxidative effects [17]. Thus, *M. balsamina* appears to have significant potential as a powerful anticancer and antioxidant agent, and this study examined its effects on cisplatin-induced kidney toxicity.

## Materials and methods

### Plant collection and identification

*Momordica balsamina* Linn. leaves were collected at the University of Limpopo, South Africa. Dr. B.A. Egan, a curator at the Larry Leach Herbarium (UNIN), performed plant identification, and voucher specimen UNIN121046 was archived.

### Plant extraction, cisplatin preparation and treatments

The leaves of *M. balsamina* were washed with distilled water and air-dried at room temperature before being ground into a powder using a blender. A total of 20 g of plant powder was extracted by boiling in 200 ml of distilled water for 10 min [18]. The extract was allowed to cool to room temperature and then filtered through Whatman No. 2 filter paper and freeze-dried. The resulting dried extract was resuspended in 1x PBS, aliquoted to stock concentrations of 100 mg/ml (w/v) and stored at -20 °C until use. Extract concentrations (200–1000 µg/ml) were selected based on preliminary cytotoxicity assays and literature on related *Momordica* species [16].

A stock solution of 1000 µg/ml (3333 µM) cisplatin (Merck, USA) was prepared with 1x PBS and stored at 4 °C until use. Cells were co-treated with cisplatin (60 µM), this concentration was selected based on preliminary concentration–response experiments (**Figure ****S1**) showing submaximal HEK-293 viability (~ 60% at 24 h).

### Phytochemical analysis of *Momordica balsamina* aqueous leaf extract

The chemical composition of MBAE was assessed using an Agilent 8890 gas chromatography (GC) system coupled with mass spectrometry (MS). Analysis was performed on a Phenomenex 5Sil MS column (30 m × 0.25 mm × 0.25 μm). A 1 µl aliquot of the extract was injected into the GC at an inlet temperature of 280 °C using helium as the carrier gas at a constant flow rate of 1.2 ml/min. The oven temperature program began at 40 °C, held for 2 min, and then increased by 9 °C/min to a final temperature of 280 °C, which was maintained for 10 min until the analysis concluded. Mass Hunter software was used to process the data acquired from the GC−MS, with chromatograms reprocessed and compounds identified through spectral matching against the NIST and Wiley library databases.

### Cell lines and culturing conditions

Human embryonic kidney 293 (HEK-293) and MDA-MB-231 cell lines were obtained from Cellonex in South Africa. HEK-293 cells were selected as a well-established in vitro model for cisplatin nephrotoxicity studies. The MDA-MB-231 cell line was selected for this study because it represent an aggressive triple-negative breast cancer subtype and has a well-characterized cisplatin response [19]. Cells were grown in minimum essential medium (MEM) (Merck, Germany), supplemented with 10% (v/v) heat-inactivated fetal bovine serum (FBS) (Capricorn Scientific, Germany), and 1% Penicillin-Streptomycin-Neomycin (PSN) (Biowest, UK) to avoid bacterial contamination. All cell lines were kept in a humidified incubator of 5% carbon dioxide (CO_2_) and 95% air at 37 °C. The cells were sub-cultured at 60–80% confluency.

### Cell counting kit-8 assay

The effects of the extract, alone or in combination with cisplatin, on the viability of HEK-293 and MDA-MB-231 cells were evaluated using the Cell Counting Kit-8 (CCK-8) assay, according to the manufacturer’s instructions (Abcam, UK). The cells (2.5 × 10^3^/well) were treated for 24–48 h with extract concentrations ranging from 200 µg/ml to 1000 µg/ml alone or in combination with 60 µM cisplatin. Following treatment, 10 µl of CCK-8 reagent was added to each well, and the cells were incubated for 2 h. The absorbance was measured at 450 nm using a Multiscan SkyHigh microplate spectrophotometer (Thermo Fisher Scientific, USA). The data obtained were expressed as a percentage of the control group. Drug-extract interactions were quantified using the mutually non-exclusive combination index (CI) method described by [20], according to the formula below:

$$\:CI=\frac{\left({D}_{MBAE}\right)comb}{{(D}_{x})MBAE}+\frac{\left({D}_{cisplatin}\right)comb}{\left({D}_{X}\right)cisplatin}+\frac{\left({D}_{MBAE}\right)comb\times\:\left({D}_{cisplatin}\right)comb}{\left({D}_{X}\right)cisplatinn\times\:\left({D}_{X}\right)cisplatin}$$

### Spheroid growth assay

A spheroid growth assay was used to assess the effects of cisplatin, alone or in combination with the extract, on HEK-293 or MDA-MB-231 spheroids as described by [21] with slight modifications. Briefly, HEK-293 or MDA-MB-231 (5 × 10^3^/well) cells were seeded in 96-well plates coated with 70 µl of UV-sterilized 1.2% (w/v) agarose gel. The cultures were grown for approximately 10 days to allow spheroid formation. The spheroids were then treated for 10 days with 60 µM of cisplatin alone or in combination with 200 µg/ml or 800 µg/ml of the extract. Fresh treatments were added every 2 days, and the spheroids were photographed to monitor their sizes under a 4x objective of a phase-contrast inverted light microscope (Zeiss, Germany). Spheroid diameters were assessed using the LC-micro software (version 5.10). The data are presented as spheroid diameters (µm) and growth rates (%) and were calculated using the following formula:

$$\:Growth\,rate\%\mathrm{=}\frac{diameter\,of\,spheroids\,on\,day\,10}{diameter\,of\,spheroid\,on\,day\,0}\times\:100$$

### CellTiter-Glo 3D cell viability assay

The effects of cisplatin, alone and in combination with the extract, on cell viability were assessed in HEK-293 and MDA-MB-231 spheroids using the CellTiter-Glo 3D cell viability assay (Promega, USA) according to the manufacturer’s instructions. MDA-MB-231 or HEK-293 spheroids were exposed to 60 µM of cisplatin alone or in combination with 200 µg/ml or 800 µg/ml of the extract for 10 days. The spheroids were transferred to an opaque-walled 96-well plate and incubated with 100 µl of CellTiter-Glo 3D reagent at room temperature for 25 min. A GloMax^®^ Discover microplate reader (Promega, USA) was used to read luminescence with an integration time of 0.5 s per well. The data are expressed as a percentage of the control group.

### Annexin V-FITC apoptosis detection assay

The mode and percentages of cell death induced by the extract, cisplatin, or their combination in HEK-293 or MDA-MB-231 cells was determined using the Muse^®^ Annexin V & Dead Cell Kit following the manufacturer’s protocol (Luminex Corporation, USA). Briefly, HEK-293 or MDA-MB-231 cells (5 × 10^3^/well) were seeded in 48-well plates and incubated overnight before being treated with 200 µg/ml to 1000 µg/ml of the extract alone or in combination with 60 µM of cisplatin for 24–48 h. After treatment, cells were harvested by trypsinization and centrifuged at 277 *× g* for 5 min. The pellets were resuspended in 100 µl of Muse Annexin V & Dead Cell Reagent and incubated in the dark for 20 min at room temperature. A Muse cell analyzer (Luminex Corporation, USA) was used to analyze the samples, and the data are expressed as graphs presenting the percentages of early apoptotic, late apoptotic, and dead cells.

### DNA damage assay

The effects of cisplatin, alone or in combination with the extract, on DNA damage induced in HEK-293 cells were evaluated using the Muse Multi-Color DNA Damage Assay Kit (Luminex Corporation, USA), according to the manufacturer’s protocol. HEK-293 cells (7 × 10^5^/well) were seeded in 25 cm^3^ cell culture flasks overnight and exposed to 60 µM of cisplatin alone or in combination with 200 µg/ml or 800 µg/ml of the extract for 24–48 h. The cells were harvested, centrifuged at 277 *× g* for 5 min, fixed, and permeabilized on ice for 10 min each. The samples were then incubated in the dark for 30 min with an antibody cocktail. Following a wash step, the pellets were resuspended in 200 µl of 1x assay buffer and analyzed using a Muse cell analyzer. The data are presented as percentages of ATM+, γH2AX+, and ATM + γH2AX co-activated cells.

### Cell cycle progression assay

The effects of cisplatin and MBAE combinatorial treatments on HEK-293 cell cycle progression were assessed at 24–48 h using the Muse Cell Cycle Kit (Luminex Corporation, USA), according to the manufacturer’s protocol. HEK-293 cells (5 × 10^3^/well) were seeded in 48-well plates and exposed to 60 µM of cisplatin only or combined with 200 µg/ml or 800 µg/ml of MBAE for 24–48 h. The cells were harvested, centrifuged for 5 min at 300 × g, washed once with 1× PBS, and then centrifuged as before. Subsequently, the cells were fixed for 3 hours at 20 °C with 70% ethanol. Fixed cells were centrifuged and washed as previously described and incubated in 200 µl of a cell cycle reagent in the dark for 30 min. Samples were analyzed using the Muse cell analyzer and presented as percentages of cell distribution.

### Western blot analysis

The effects of cisplatin, alone or in combination with the extract, on the expression profiles of Bcl-2-associated X protein (Bax), B-cell lymphoma-2 (Bcl-2), tumor protein 53 (TP53/p53), protein 21 (p21), and poly (ADP-ribose) polymerase 1 (PARP1) were assessed using western blotting as previously described [22]. HEK-293 cells (0.7 × 10^6^/well) were seeded in 25 cm^3^ cell culture flasks overnight and treated for 48 h with 60 µM cisplatin alone or in combination with 200 µg/ml or 800 µg/ml of the extract for 48 h. The cells were harvested by trypsinization, centrifuged for 5 min at 571 *× g*, and washed with 1x PBS. The pellets were resuspended and incubated for 1 h with 150 µl of RIPA buffer [50 mM Tris-HCl (pH 7.4), 150 mM NaCl, 2 mM EDTA, 0.1% SDS, 1% Triton X-100, 1% sodium deoxycholate, and 1x protease inhibitor cocktail] and subjected to sonication followed by centrifugation at 4000 *× g* at 4 °C for 15 min. Protein quantification was performed using the BCA protein assay Kit (Sigma-Aldrich, Germany) according to the manufacturer’s instructions. Aliquots containing 29 µg of protein were resolved on 12% sodium dodecyl sulfate polyacrylamide gels. Proteins were electro-blotted onto Polyvinylidene difluoride (PVDF) membranes at 200 mA for 1 h and blocked for another hour with blocking buffer 3% (w/v) bovine serum albumin in PBS containing 0.05% (v/v) Tween 20. The membranes were incubated with HRP-labeled mouse antibodies against 𝞫-actin (1:1000), rabbit primary antibodies against PARP-1 (1:500) or p21 (1:500), and mouse primary antibodies against p53 (1:500), Bax (1:200), or Bcl-2 (1:200) at 4 °C on a rocking platform overnight. Following incubation, each membrane was washed 3 times with 10 ml of wash buffer (0.05% PBS-Tween 20) and incubated for 2 h with HRP-conjugated secondary antibodies (1:20 000). The membranes were washed again and incubated with a chemiluminescent reagent mixture (Thermo Fisher Scientific, USA) for 5 min before being photographed using the G: BOX imaging system with GeneSystem software (version 1.8.6.0) (Syngene, UK). Band sizes were quantified using ImageJ software (version 1.52a) and expressed as the fold change relative to β-actin expression.

### Statistical analysis

Statistical variations among controls and treatments were calculated using GraphPad Prism software (version 8.4.2) by ordinary one-way analysis of variance (nonparametric or mixed), followed by Dunnett’s comparison test or Tukey’s multiple comparisons test. The data were expressed as the standard mean of error (SEM) of three independent experiments performed in duplicate and p ˂ 0.01 indicates a significant difference compared with the untreated control or cisplatin-only treatment.

## Results

### Phytochemical composition of MBAE

Twenty-six tentatively identified compounds are presented in Table 1 categorized by phytochemical class, IUPAC name, molecular formula, retention time, and match factor. These compounds belong to various chemical classes, including flavonoids and phenolic compounds, lactones and furanone, alkaloids and heterocyclic compounds, fatty acid and lipid derivatives, piperidine and morpholine derivatives, nitriles, and terpenoids. Some well-known compounds identified in MBAE include N-[2-(4-Morpholinyl)propyl]-4-oxo-2,3,4,5-tetrahydro-1,5-benzothiazepine-7-carboxamide, flavonol 3’,4’,5,7-OH,3-O-araglucoside, and arginine. Compounds known for their bioactivity, such as guaiacol, flavonol 3’,4’,5’,7-OH,3-O-araglucoside, and lactone G, were also identified. On the basis of their low match factors, compounds most likely novel in *M. balsamina* were also identified, these include flavonol 3’,4’,5,7-OH,3-O-araglucoside (52), 5-(hydroxymethyl)dihydrofuran-2(3 H)-one (58), pentahalide, 2-(dimethylamino)-3-methyl-N-[7-(2-methylpropyl)-5,8-dioxo-3-phenyl-2-oxa-6,9-diazabicyclo[10.2.2]hexadeca-10,12,14,15-tetraen-4-yl]-, [3R-[3R,4 S(2 S*,3R*),7 S*]]-** (51), 2,4,6,8-Tetradecatetraenoic acid, 9a-(acetyloxy)-1a,1b,4,4a,5,7a,7b,8,9,9a-decahydro-4a,7b-dihydroxy-3-(hydroxymethyl)-1,1,6,8-tetramethyl-5-oxo-1 H-cyclopropa [3, 4]benz[1,2-e]azulen-9-yl ester (50) and 1b,4a-Epoxy-2 H-cyclopenta [3, 4]cyclopropa [8, 9]cycloundec[1,2-b]oxiren-5(6 H)-one 7-(acetyloxy)decahydro-2,9,10-trihydroxy-3,6,8,8,10a-pentamethyl- (51).

**Table 1:** Characterization of phytochemical constituents in *Momordica balsamina* aqueous extract

Phytochemical class	IUPAC name	Chemical formulae	Retention time (min)	Match factor
Flavonoids and Phenolic Compounds	Flavonol 3’,4’,5,7-OH,3-O-araglucoside	C_26_H_30_O_16_	10.77	52
	Guaiacol	C_7_H_8_O_2_	10.08	63
	Maltol	C_6_H_6_O_3_	10.47	57
	4 H-Pyran-4-one, 2,3-dihydro-3,5-dihydroxy-6-methyl-	C_6_H_8_O_4_	11.00	96
Lactones and Furanones	2,4-Dihydroxy-2,5-dimethyl-3(2 H)-furan-3-one	C_6_H_8_O_4_	8.308	78
	Furaneol	C_6_H_8_O_3_	9.79	79
	2(3 H)-Furanone, dihydro-5-propyl-	C_7_H_12_O_2_	12.06	58
	(S)-5-Hydroxymethyl-2[5 H]-furanone	C_5_H_6_O_3_	11.53	62
	5-(Hydroxymethyl)dihydrofuran-2(3 H)-one	C_5_H_8_O_3_	11.72	58
	Lactone G	C_5_H_8_O_4_	15.01	65
Alkaloids and Heterocyclic Compounds	1,4-Diazabicyclo[2.2.2]octane	C_6_H_12_N_2_	8.692	67
	1,3-Diazacyclooctane-2-thione	C_6_H_12_N_2_S	13.14	69
	N-[2-(4-Morpholinyl)propyl]-4-oxo-2,3,4,5-tetrahydro-1,5-benzothiazepine-7-carboxamide	C_17_H_23_N_3_O_3_S	11.91	66
	Pyrido[2,3-d]pyrimidine-2,4(1 H,3 H)-dione, 6,7-dichloro-5-[(1-ethylpyrrolidin-2-yl)methylamino]-1,3-dimethyl-	C_16_H_21_Cl_2_N_5_O_2_	9.367	60
	1,3-Diazacyclooctane-2-thione	C_6_H_12_N_2_S	13.14	69
	Pentanamide, 2-(dimethylamino)-3-methyl-N-[7-(2-methylpropyl)-5,8-dioxo-3-phenyl-2-oxa-6,9-diazabicyclo[10.2.2]hexadeca-10,12,14,15-tetraen-4-yl]-, [3R-[3R*,4 S*(2 S*,3R*),7 S*]]-	C_31_H_42_N_4_O_4_	13.85	51
	Aralionine	C_34_H_38_N_4_O_5_	9.53	61
	2-Azabicyclo[3.3.0]octane, 3-(hydroxydiphenylmethyl)-, cis-	C_20_H_23_NO	18.84	51
Fatty Acid and Lipid Derivatives	Decanedioic acid, 3,8-dioxo-, dimethyl ester	C_12_H_18_O_6_	14.55	56
	1,2,3-Propanetriol, 1-acetate	C5H10O4	12.49	68
Piperidine and Morpholine Derivatives	N-[4-(4-Chlorophenyl)isothiazol-5-yl)-1-methylpiperidin-2-imine	C_15_H_16_ClN_3_S	15.95	51
	Tridemorph	C_19_H_39_NO	8.782	57
Nitrile	Butane-1,1-dicarbonitrile, 1-cyclohexyl-3-methyl-	C_13_H_20_N_2_	10.21	62
Terpenoids	2,4,6,8-Tetradecatetraenoic acid, 9a-(acetyloxy)-1a,1b,4,4a,5,7a,7b,8,9,9a-decahydro-4a,7b-dihydroxy-3-(hydroxymethyl)-1,1,6,8-tetramethyl-5-oxo-1 H-cyclopropa[3,4]benz[1,2-e]azulen-9-yl ester, [1aR-(1a.alpha.,1b.beta.,4a.beta.,7a.alpha.,7b.alpha.,8.alpha.,9.beta.,9a.alpha.)]-	C_36_H_48_O_8_	37.92	50
	2,2,6-Trimethyl-12-oxabicyclo[8.2.1]trideca-3,6,10(13)-triene-5,11-dione	C_15_H_18_O_3_	13.39	60
	1b,4a-Epoxy-2 H-cyclopenta[3,4]cyclopropa[8,9]cycloundec[1,2-b]oxiren-5(6 H)-one, 7-(acetyloxy)decahydro-2,9,10-trihydroxy-3,6,8,8,10a-pentamethyl-	C_22_H_32_O_8_	17.68	51

### Effect of MBAE extract and combination treatment with cisplatin on HEK-293 cell viability

**Fig. 1:**
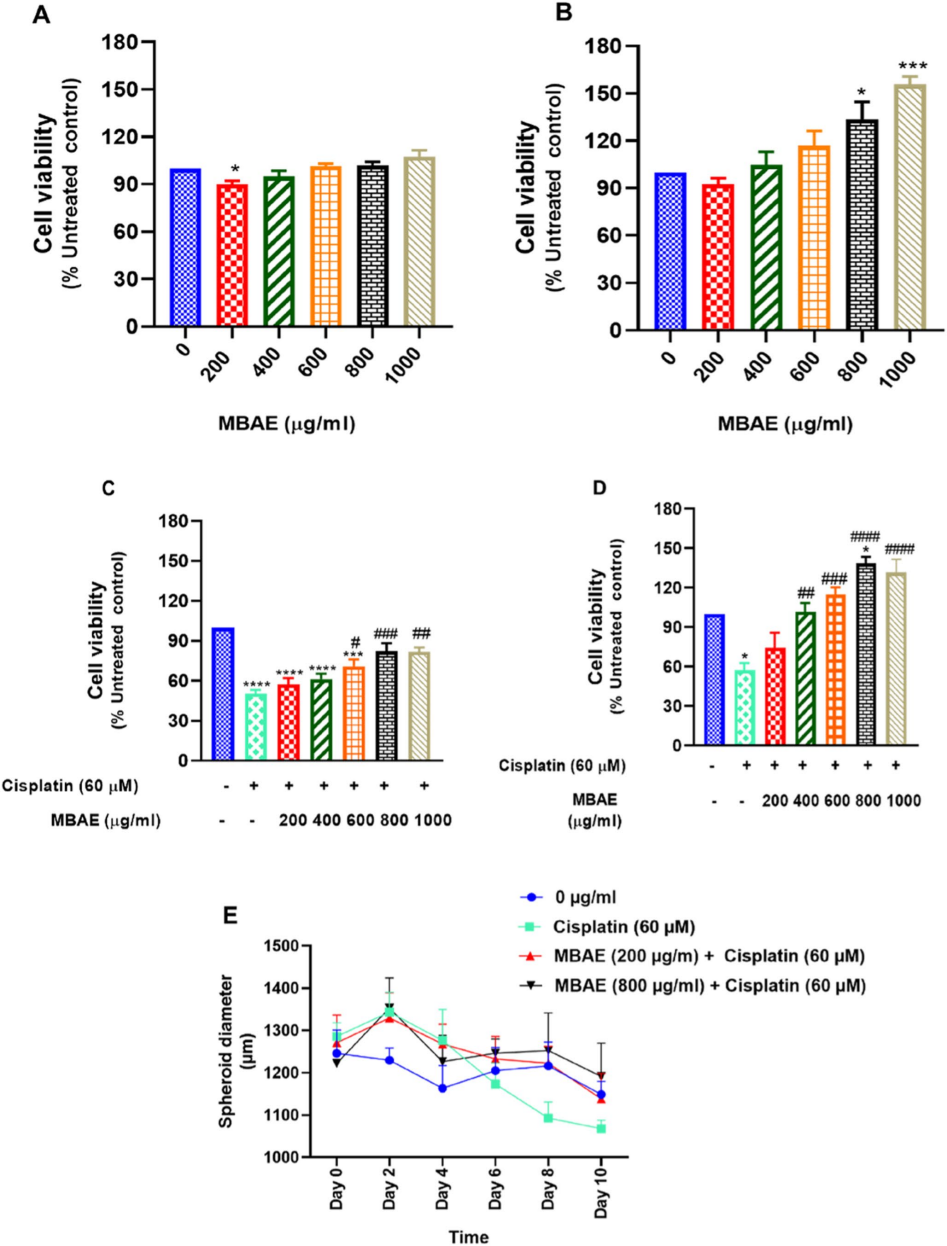

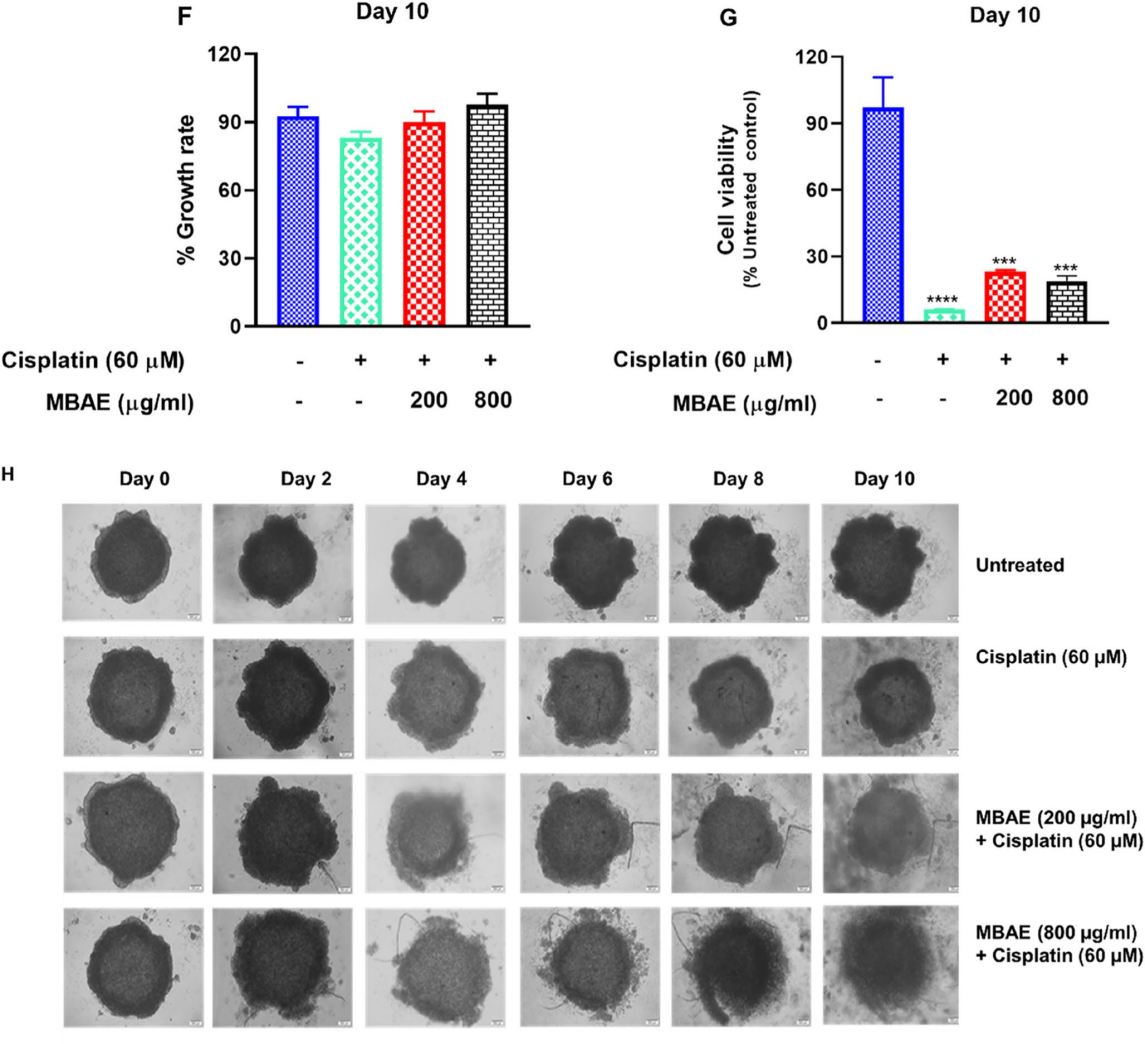
Effect of cisplatin and MBAE combinatorial treatments on HEK-293 cell and spheroid viability, and on spheroid diameters and growth rates. Percentage viability of HEK-293 treated with MBAE alone for 24 h (**A**) and 48 h (**B**) or MBAE in combination with cisplatin for 24 h (**C**) and 48 h (**D**). Spheroid diameters measured in micrometres (**E**), percentage growth rates of spheroids (**F**), spheroids viability (**G**) and spheroid morphology (**H**). Data are presented as mean ± standard error of the mean (SEM) from three independent experiments performed in duplicate. Data were expressed as the standard error of the mean (SEM) from three independent experiments conducted in duplicates. *p ˂ 0.01 and ***p ≤ 0.0001, ****p < 0.0001 indicate significant differences from the untreated control. #*p* < 0.01, ##*p* ≤ 0.001, ###*p* ≤ 0.001, and ####*p* < 0.0001 denote significant differences relative to cisplatin treated cells

Cells treated with MBAE showed a time- and concentration-dependent increase in viability compared with the untreated control, except for the 200 µg/ml treatment, which reduced cell viability at 24 (*p* < 0.01) (Fig. 1A) and 48 h (Fig. 1B) of treatment. Treatment with 60 µM of cisplatin significantly decreased HEK-293 cell viability at 24 h (p ˂ 0.0001, Fig. 1C) and 48 h (p ˂ 0.01; Fig. 1D), relative to the untreated control. Combining cisplatin with the extract decreased cell viability percentages after 24 h of treatment compared with the untreated control (Fig. 1C). However, cell viability was increased in a concentration-dependent manner relative to cisplatin-only treatment, significantly from 600 µg/ml to 1000 µg/ml of co-treatment with the extract. At 48 h, co-treatment with 800 µg/ml of the extract increased cell viability significantly (p ˂ 0.01) compared with the untreated control (Fig. 1D). Compared with cisplatin-only treatment, the combinatory treatments significantly enhanced cell viability in a time- and concentration-dependent manner, significantly at 400 µg/ml (*p* ≤ 0.001), 600 µg/ml (*p* ≤ 0.001), 800 µg/ml (*p* < 0.0001) and 1000 µg/ml (*p* < 0.0001) of co-treatment.

### Effect of MBAE and cisplatin combination treatments on HEK-293 spheroids diameters, growth rates, and viability

The diameters of spheroids treated with cisplatin alone or in combination with the extract increased from day 0 to day 4 compared with untreated spheroids (Fig. 1E). However, a significant reduction in spheroid diameter was observed between days 4 and 10 in the cisplatin-only treated spheroids, decreasing from 1276.25 μm to 1068.01 μm relative to untreated spheroids. Meanwhile, co-treatment showed a progressive decrease in diameter from days 2 to 10, with sizes of approximately 1138.38 μm and 1191.5 μm, respectively, although remaining larger than those of spheroids treated with cisplatin alone. Moreover, spheroids exposed to a combination of cisplatin and 200 µg/ml of the extract were smaller than those of untreated spheroids, whereas those co-treated with 800 µg/ml of the extract were larger. This implies that the latter displayed a higher level of protection against cisplatin-induced toxicity. Growth rate analysis (Fig. 1F) revealed that spheroids treated solely with cisplatin exhibited reduced growth rates compared with the untreated control. No significant differences were observed between untreated spheroid growth rates and co-treated spheroids. Although statistically insignificant, the combinatory treatments enhanced spheroid growth rates in a concentration-dependent manner compared to spheroids treated with cisplatin alone. In addition, cisplatin treatment significantly decreased the viability percentages compared with untreated spheroids (*p* < 0.0001) (Fig. 1G). Similarly, the combination treatments decreased the viability percentages relative to the untreated spheroids (*p* < 0.0001). However, these percentages were markedly increased compared with cisplatin-only treated spheroids.

### Cell death induced by MBAE and combination treatment with cisplatin in HEK-293 cells

**Fig. 2:**
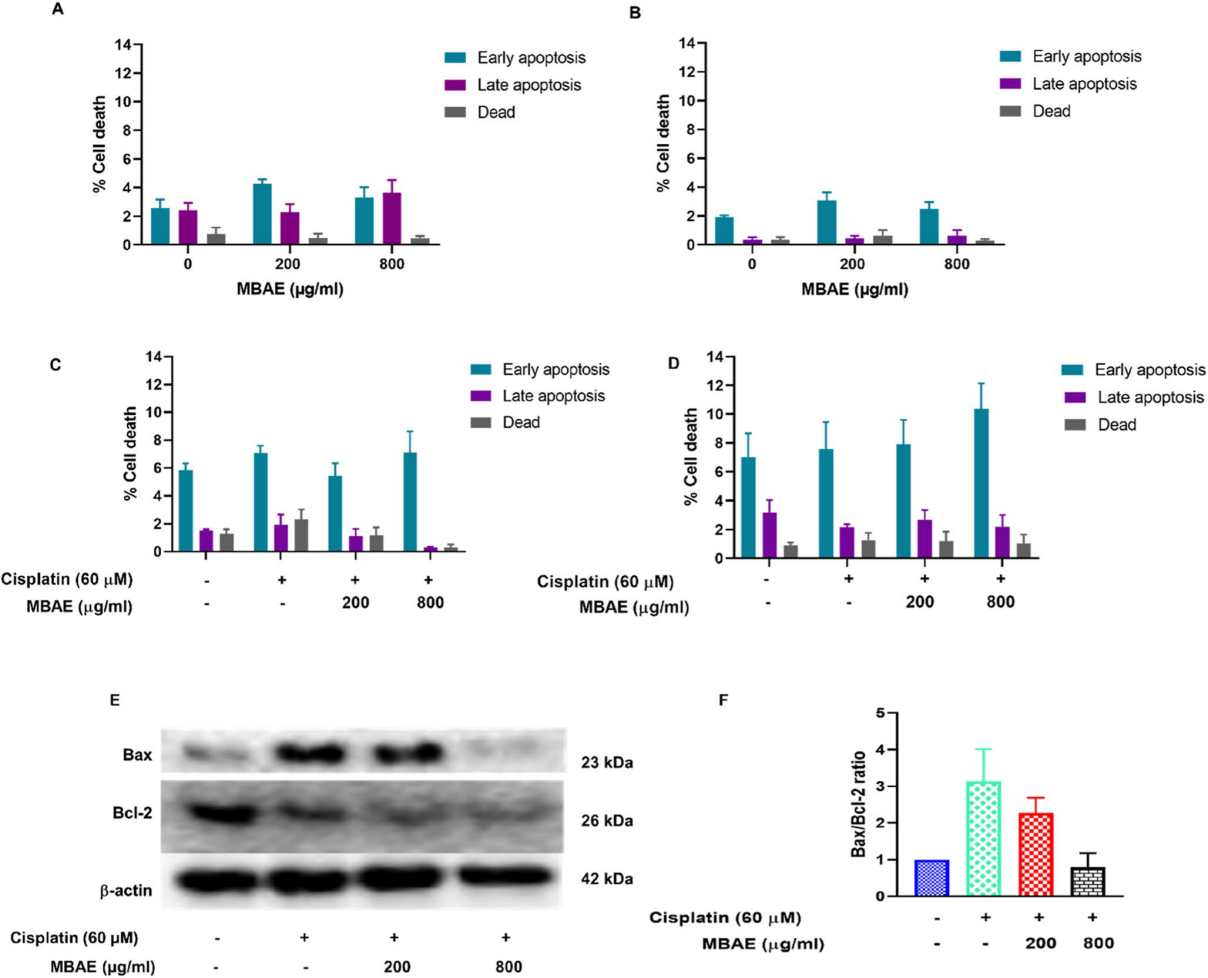
Induction of apoptosis by MBAE alone or in combination with cisplatin. The mode of death induced by MBAE at 24 h (**A**) and 48 h (**B**) and combinatorial treatment with cisplatin at 24 h (**C**) and 48 h (**D**) in HEK-293 cells. Expression of Bax and Bcl-2 was assessed, and bands were visualized and photographed using GeneSys software (**E**). Band intensities were quantified using ImageJ software and represented as the ratios of Bax to Bcl-2 (**F**). The data are presented as the standard error of the mean (SEM) from three independent experiments, each conducted in duplicates. The absence/presence of cisplatin is denoted by -/+

Treatment with 200 µg/ml or 800 µg/ml of the extract had no significant effect on the percentages of early apoptotic, late apoptotic, and dead cells at 24 (Fig. 2A) and 48 h (Fig. 2B) compared with the untreated control. A similar response was observed following the combinatorial treatment of cisplatin with the extract at 24 (Fig. 2C) and 48 h (Fig. 2D). The combination treatment increased the percentage of early apoptotic cells over late apoptotic or dead cells in a concentration-dependent manner, especially at 48 h of treatment (Fig. 2D).

### Effect of the combination treatment of cisplatin and MBAE on Bax and Bcl-2 protein expression and Bax/Bcl-2 ratios

Cisplatin treatment downregulated Bcl-2 and upregulated Bax protein expression compared with the untreated control (Fig. 2E). In contrast, combinatorial treatment with 200 µg/ml or 800 µg/ml of the extract downregulated Bcl-2 protein expression compared with the untreated and cisplatin-only treatments. The expression of Bax protein was upregulated following combinatorial treatment with 200 µg/ml of the extract and downregulated following treatment with 800 µg/ml of the extract relative to the untreated control. Compared with cisplatin-only treatment, the expression of Bax was concentration-dependently downregulated. The Bax/Bcl-2 ratio increased in cisplatin-only treated cells; however, compared with the cisplatin-only treatment, the combinatory treatments reduced the Bax/Bcl-2 ratios in a concentration-dependent manner (Fig. 2F). Furthermore, combinatorial treatment with 800 µg/ml of the extract decreased the Bax/Bcl-2 ratios compared with the untreated control, although the difference was not statistically significant.

### Effect of MBAE on cisplatin-induced DNA damage

**Fig. 3:**
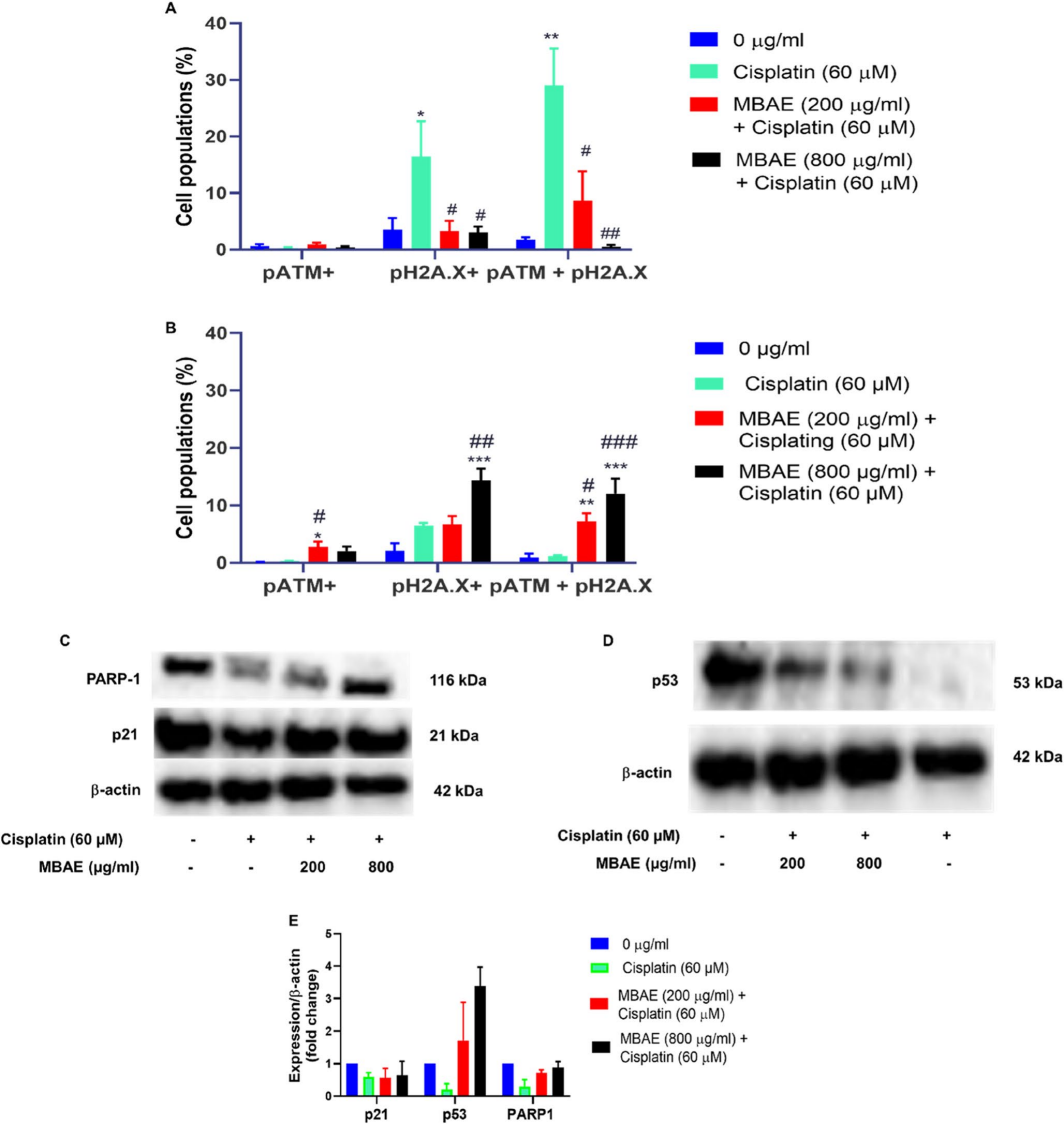
Effect of cisplatin and MBAE combinatorial treatments on pATM and γH2AX levels, and PARP1, p21 and p53 PR in HEK-293 cells. HEK-293 cells were treated for 24 h (**A**) and 48 h (**B**). PARP1 and p21 expression profile (**C**), p53 expression profile (**D**). Band intensities quantified with ImageJ software and presented as fold change relative to β-actin expression (**E**). Data are presented as the standard error of the mean (SEM) from three independent experiments conducted in duplicates. *p ˂ 0.01, **p ˂ 0.001, and ***p ≤ 0.0001, denote significant differences from the untreated control. #p ˂ 0.01, ##p ˂ 0.001, and ###*p* ≤ 0.0001 indicate significant differences compared to cells treated only with cisplatin. -/+ denotes the absence or presence of cisplatin

Cisplatin treatment significantly increased the percentage of pH2A.X- and pATM + as well as pATM+pH2A.X-positive cells at 24 h compared with the untreated control. Comparatively, the combination treatment with the extract induced a significant (p ˂ 0.001 and *p* ≤ 0.0001) and concentration-dependent decrease in pH2A.X- and pATM+pH2A.X-positive cells compared with the untreated control and cisplatin-only treatment (Fig. 3A). Cisplatin treatment had no significant effect on the percentages of pATM+, pH2A.X+, and co-activated cells at 48 h (Fig. 3B) compared with the untreated control. Combination treatment with the extract increased pATM+ (*p* < 0.0001), pH2A.X+ (*p* < 0.001), and co-activated (*p* < 0.01) cell percentages relative to untreated cells and cisplatin-only treatment. These findings imply that while extract supplementation decreases cisplatin-mediated DNA damage in 24 h, extended exposure may increase DNA damage to initiate DDR.

### Effect of combination treatment with cisplatin and MBAE on p53, p21, and PARP1 protein expression

Cisplatin treatment downregulated p53 protein expression relative to the untreated control (Fig. 3C). In contrast, the combinatory treatments upregulated p53 protein expression comparable to cisplatin-only treatment. Notably, quantification of p53 protein expression revealed a concentration-dependent increase relative to both the untreated control and cisplatin-only treatment (Fig. 3E). Additionally, the cisplatin-only treatment downregulated p21 protein expression compared with the untreated control (Fig. 3E), whereas the combination treatment upregulated p21 protein expression in a concentration-dependent manner relative to the cisplatin-only treatment (Fig. 3E). The expression of protein PARP1 was downregulated by cisplatin-only treatment relative to the untreated control (Fig. 3D); however, combining cisplatin with the extract upregulated PARP1 protein expression in a relatively concentration-dependent manner (Fig. 3D). These data suggest that the extract potentiates the PARP1/p53 pathway to repair cisplatin-induced DNA damage in HEK-293 cells.

### Effect of combination treatment with cisplatin and MBAE on cell cycle progression

**Fig. 4:**
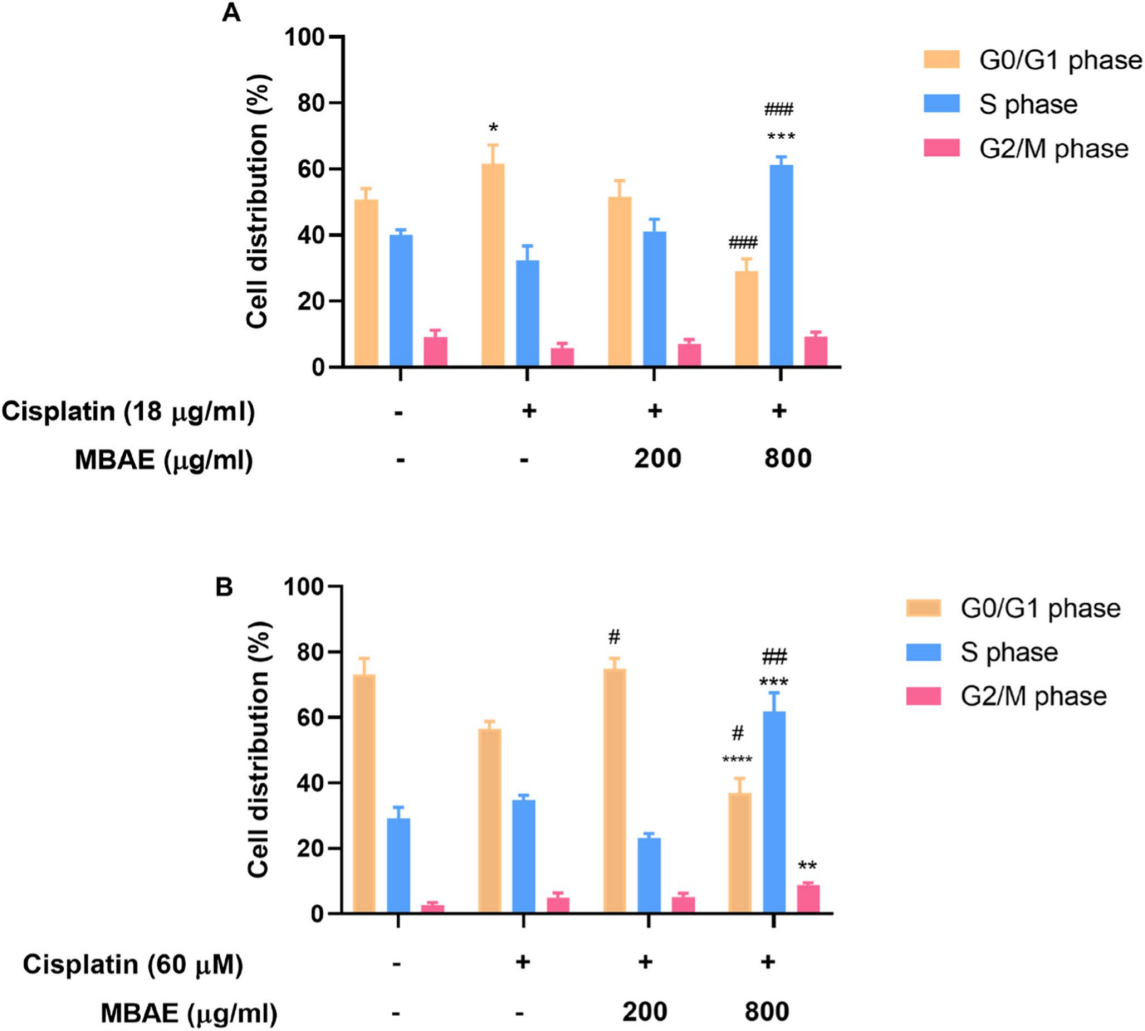
Effect of cisplatin and MBAE combinatorial treatments on HEK-293 cell cycle distribution at 24 (**A**) and 48 h (**B**). Data were expressed as the standard error of the mean (SEM). *p < 0.01, **p < 0.001, ***p ≤ 0.0001, and ****p < 0.0001 denote significant differences compared with the untreated control. #p ˂ 0.01, ##p ˂ 0.001 and ###*p* ≤ 0.001 indicate significant differences compared with cisplatin-only treated cells

Cisplatin treatment for 24 h resulted in a significant (*p* < 0.01) increase in G0/G1-phase cell populations, with no significant effects on S-phase or G2/M-phase populations (Fig. 4A). Combinatorial treatment with 200 µg/ml of the extract did not affect cell cycle progression. In contrast, combinatorial treatment with 800 µg/ml of the extract decreased G0/G1-phase populations significantly (*p* ≤ 0.0001) compared with cisplatin-only treatment and increased S-phase populations compared with both the untreated control (*p* ≤ 0.0001) and cisplatin-only treatment (*p* ≤ 0.0001). Cisplatin treatment did not affect cell cycle progression at 48 h (Fig. 4B). However, combinatorial treatment with 200 µg/ml of the extract resulted in a significant (*p* < 0.01) increase in G0/G1-phase populations compared with cisplatin-only treatment but did not affect S-phase and G2/M-phase cell populations. Combinatorial treatment with 800 µg/ml of extract significantly decreased the G0/G1-phase populations relative to the untreated cells (*p* < 0.0001) and cisplatin-only treatment (*p* < 0.01). In addition, S-phase populations were significantly increased relative to untreated cells (*p* ≤ 0.0001) and cisplatin-only treatment (*p* < 0.001). Furthermore, G2/M-phase populations were increased compared with untreated cells (p ˂ 0.001). Taken together, these findings suggest that the extract halts cisplatin-challenged HEK-293 cell cycle progression at the G0/G1 and S-phases to induce DNA repair.

### Effect of MBAE and cisplatin treatment on MDA-MB-231 cell viability

**Fig. 5:**
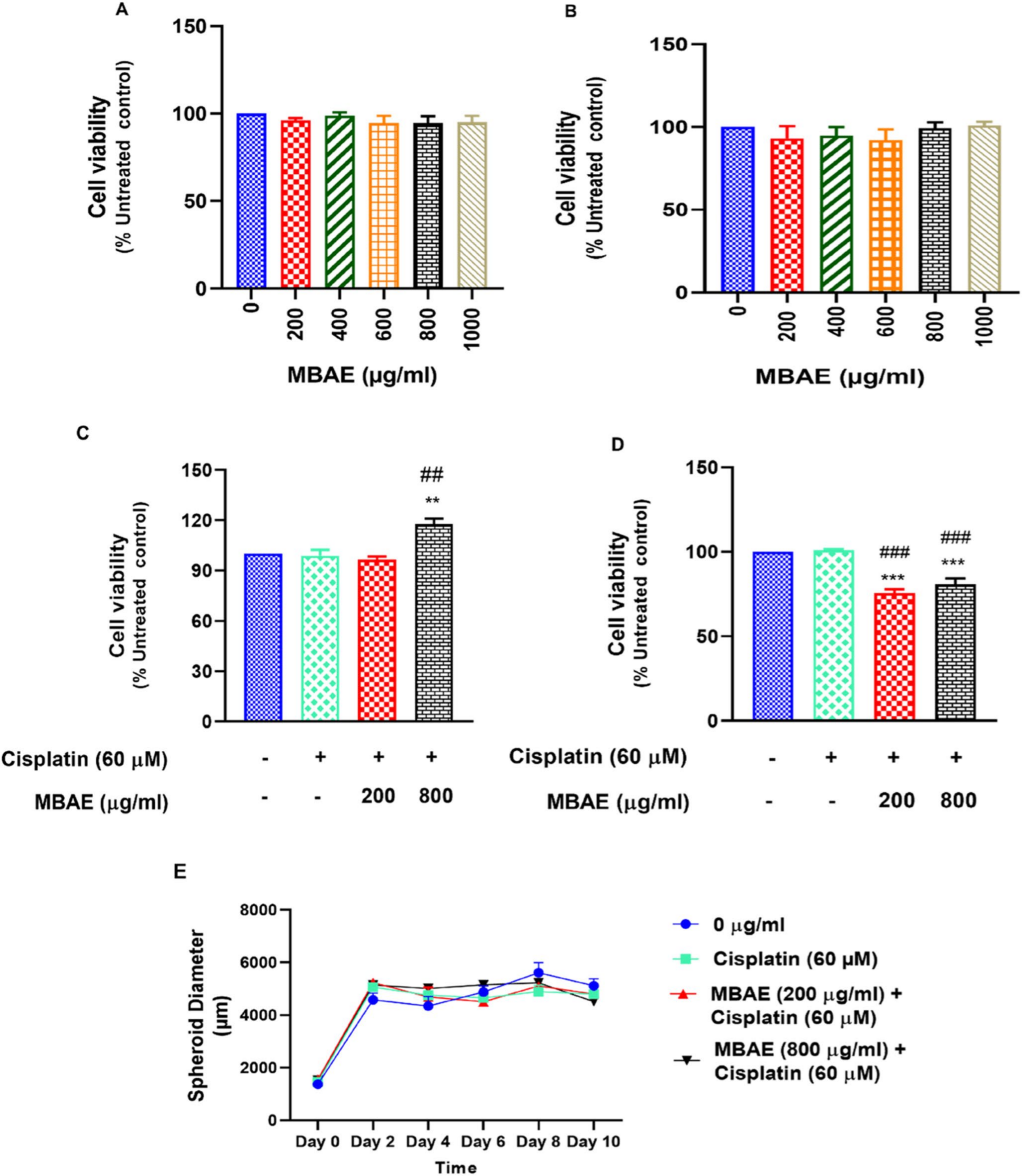

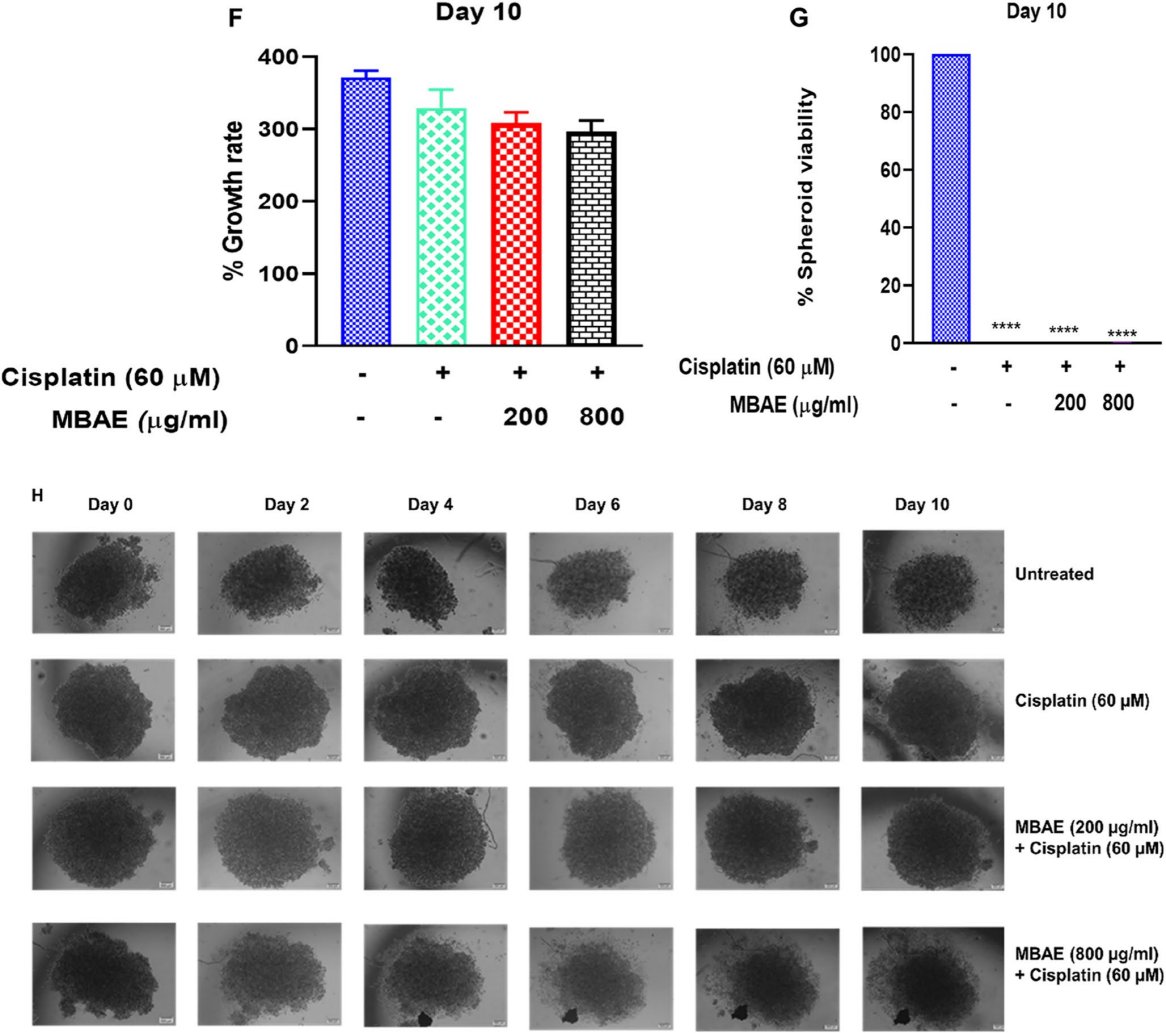
Effect of cisplatin and MBAE combinatorial treatments on MDA-MB-231 cell and spheroid viability, and on spheroid diameters and growth rates. Percentage viability of MDA-MB-231 cell treated with MBAE alone for 24 h (**A**) and 48 h (**B**) or MBAE in combination with cisplatin for 24 h (**C**) and 48 h (**D**). Spheroid diameters measured in micrometers (**E**), percentage growth rates of spheroids (**F**), spheroid viability (**G**) and spheroid morphology (**H**). Data were expressed as the standard error of the mean (SEM) from three independent experiments performed in duplicate. **p ≤ 0.001, ***p ≤ 0.0001 and ****p < 0.0001 denote significant variation from the untreated control, whereas ##*p* ≤ 0.001 and ###*p* ≤ 0.001 denote significant variations from cisplatin-only treated cells, respectively

Treatment of MDA-MB-231 cells with MBAE at concentrations ranging from 200 µg/ml to 1000 µg/ml did not cause any significant change in cell viability at 24 and 48 h (Fig. 5A and B). Similarly, cisplatin treatment for 24 h did not affect cell viability at both time points (Fig. 5C and D). Similarly, combination treatment with 200 µg/ml of the extract for 24 h had no significant effect on cell viability compared with the untreated control and cisplatin-only treatment. In contrast, combinatorial treatment with 800 µg/ml of the extract increased cell viability significantly (*p* ≤ 0.001) compared with the untreated control and cisplatin-only treatment groups (Fig. 5C). At 48 h (Fig. 5D), combination treatment with 200 µg/ml or 800 µg/ml of the extract decreased MDA-MB-231 cell viability significantly (*p* ≤ 0.001) compared with the untreated control and cisplatin-only treated cells.

### Effects of MBAE and cisplatin combination treatment on MDA-MB-231 spheroid diameter, growth rate, and viability

The diameters of spheroids treated with 60 µM cisplatin increased compared with those of untreated spheroids from days 2 to 4. From days 6 to 10, the diameters of spheroids treated with 60 µM cisplatin or a combination of 60 µM cisplatin and 200 µg/ml of the extract decreased compared with those of untreated spheroids. Furthermore, treatment with 60 µM cisplatin and 200 µg/ml of the extract resulted in decreased spheroid diameters on day 6 (± 4,509.62 μm) compared with untreated and cisplatin-only treated spheroids. By day 10, spheroids treated with cisplatin and 800 µg/ml displayed decreased diameters (± 451.98 μm) compared to all other treatments (Fig. 5E). The assessment of spheroid growth rate (Fig. 5F) showed that treatment with 60 µM cisplatin decreased the percentage growth rate compared with the untreated control. Treatment with 60 µM cisplatin and 200 or 800 µ/ml of the extract further decreased the percentage growth rate compared to both the untreated control and cisplatin-only treatment. Furthermore, treatment of MDA-MB-231 spheroids with 60 µM cisplatin significantly (p ˂ 0.0001) decreased spheroid viability compared to untreated spheroids (Fig. 5G). Spheroids exposed to 60 µM cisplatin in combination with 200–800 µg/ml of the extract further decreased spheroid viability in a concentration-dependent manner compared to untreated spheroids (*p* < 0.0001) and cisplatin-only treated spheroids, inferring that the extract potentially enhances the toxic effects of cisplatin on MDA-MB-231 cells.

### Mode of cell death induced by MBAE and combination treatment with cisplatin in MDA-MB-231 cells

**Fig. 6:**
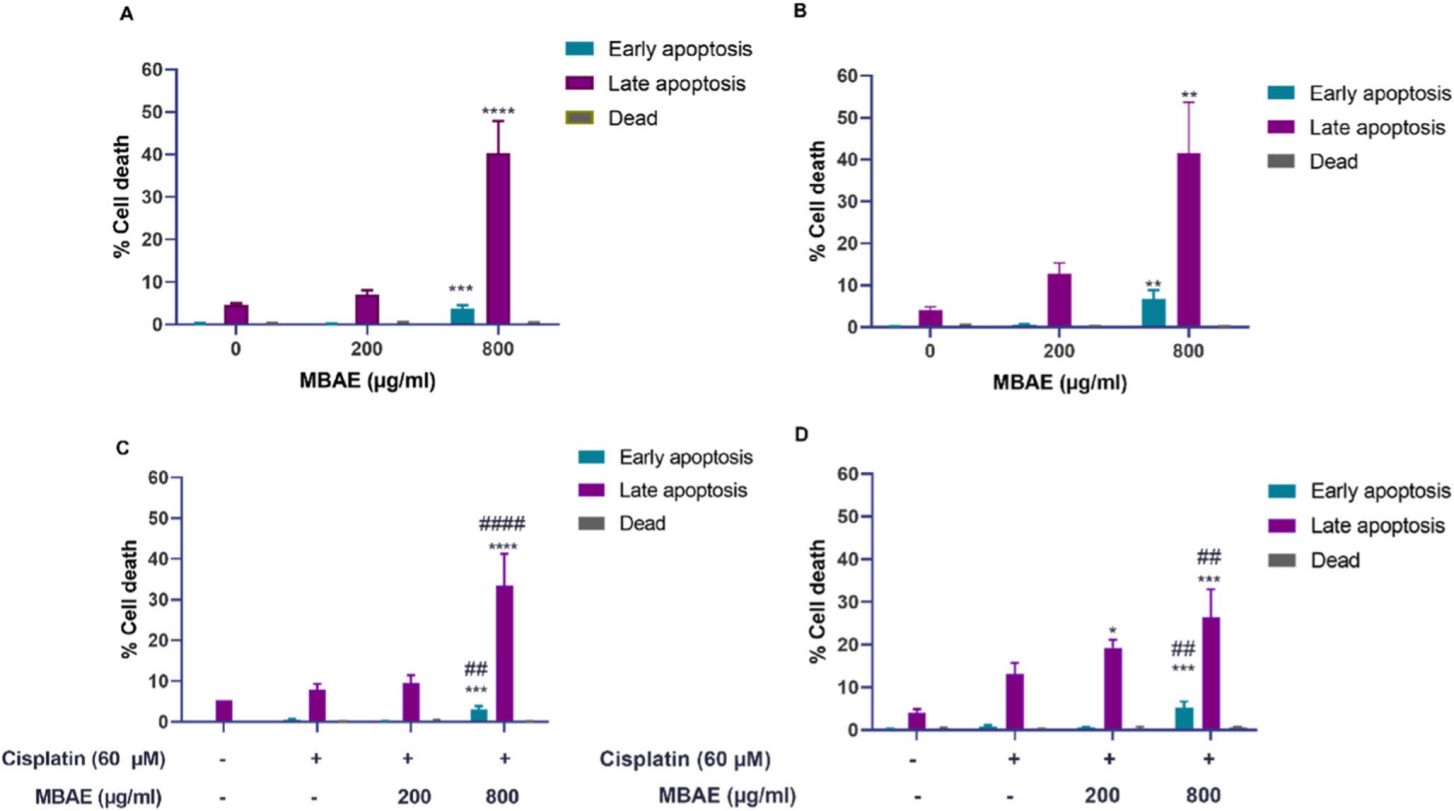
Induction of apoptosis by MBAE alone or in combination with cisplatin. The mode of death induced by MBAE extract at 24 h (**A**) and 48 h (**B**) and combinatorial treatment with cisplatin at 24 h (**C**) and 48 h (**D**) in MDA-MB-231 cells. Data were expressed as the standard error of the mean (SEM) of three independent experiments performed in duplicate. *p ˂ 0.01 indicates significant differences from the untreated control. *p < 0.01, **p < 0.001, ***p ≤ 0.0001 and ****p < 0.0001 denotes significant variation from the untreated control whereas ##*p* < 0.001 and ####*p* < 0.0001 denote significant differences from the untreated control

*Momordica balsamina* aqueous leaf extract increased the percentage of late apoptotic cells in a concentration-dependent manner compared to the untreated control at 24 and 48 h (Fig. 6A and B). Notably, treatment with 800 µg/ml significantly increased the percentages of early and late apoptosis compared with the untreated control at 24 h (*p* ≤ 0.0001 and *p* < 0.0001) and 48 h (*p* < 0.001). Treatment with cisplatin increased the percentages of late apoptotic cells at both time points compared to the untreated control (Fig. 6C and D), although not statistically significant. Combination treatment with the extract for 24 h (Fig. 6C) further increased the percentage of late apoptotic cells in a concentration-dependent manner. Notably, the percentages of early and late apoptotic cells were significantly increased by combination treatment with 800 µg/ml of MBAE compared to the untreated control (*p* ≤ 0.001, and *p* < 0.0001) and cisplatin-only treatment (*p* < 0.001 and *p* < 0.0001). At 48 h (Fig. 6D), combination treatment with 200 µg/ml (*p* < 0.01) or 800 µg/ml (*p* ≤ 0.0001) of the extract resulted in a concentration-dependent increase in the percentages of late apoptotic cells compared to the untreated control. Additionally, the percentages of late apoptotic cells were significantly increased (*p* < 0.01) by combination treatment with 200 µg/ml of the extract compared to cisplatin-only. Moreover, combination treatment with 800 µg/ml increased the percentages of early apoptotic cells significantly compared to the untreated control (*p* ≤ 0.0001) and the cisplatin-only treatment (*p* < 0.001).

## Discussion

Cisplatin-induced nephrotoxicity affects 26%−36% of patients receiving cisplatin-based treatments [17]. Intracellular stressors, including DNA damage and mitochondrial dysfunction, have been identified as mechanisms through which cisplatin exerts its toxicity in cancerous and noncancerous cells, triggering stress responses, such as cell cycle halting, apoptotic cell death induction, and inflammatory responses [23]. In clinical practice, cisplatin is used along with several other agents; however, this approach does not lessen its toxicity in kidney cells. As a result, the search for novel strategies to alleviate cisplatin-induced kidney damage has become more rigorous. Agents that induce DNA repair mechanisms and improve mitochondrial function may prove helpful in developing therapeutic interventions that may effectively retard the progression of cisplatin-induced renal pathology [24, 25]. This study aimed to elucidate potential protective mechanisms of the MBAE, a plant referred to as a “gift of nature” due to its wide range of nutraceutical and medicinal properties [24], against cisplatin-induced kidney damage. The findings of this study suggest that MBAE enhances the HEK-293 DNA damage response (DDR) by upregulating the PARP1/p53 signaling pathway. The extract further decreases cisplatin-associated intrinsic apoptosis by suppressing the Bax/Bcl-2 ratio. Moreover, extract supplementation enhanced the anticancer activity of cisplatin, as shown by reduced viability and increased apoptosis induced in MDA-MB-231 2D and 3D models. Overall, this study demonstrated for the first time the role of the *M. balsamina* aqueous extract in reducing cisplatin toxicity in renal cells by enhancing DDR and reducing the occurrence of cell death.

The identification of compounds present in plant extracts allows researchers to understand their chemical composition and attribute specific biological activities to the phytocompounds. Studies have been conducted to determine the phytochemical profiles of extracts of *M. balsamina* leaves [15, 16, 26] and fruit pulps [27]. The phytochemical composition in these studies shows significant overlap with the compounds identified in the current analysis (Table 1) while also demonstrating key differences. All reviewed studies consistently reported the presence of flavonoids and phenolic compounds, including quercetin and kaempferol glycosides, which align with the flavonol derivatives listed in the current study, including flavonol 3’,4’,5,7-OH,3-O-araglucoside, guaiacol, and maltol. Similarly, the terpenoid compounds identified in the table are congruent with triterpenoids reported in previous literature, particularly cucurbitane-type derivatives [15]. While the current study identified several alkaloids, lactones, furanones, and fatty acid derivatives, including aralionine, furaneol, and decanedioic acid 3,8-dioxo- and dimethyl esters, these constituents were not explicitly reported in the comparative analysis studies. This variation reflects methodological differences, i.e., the use of comprehensive untargeted metabolomic profiling in the present work compared with the more targeted UHPLC-qTOF-MS and chemometric approaches used in prior research. Furthermore, differences in plant parts (e.g., leaves against fruit pulp) and geographical origin also contribute to the phytochemical diversity observed. Compounds such As maltol have been studied for their vast biological activities. A study by [28] investigated the effects of maltol on cisplatin-induced nephrotoxicity and revealed that maltol induced significant anti-apoptotic effects by decreasing Bax/Bcl-2 ratios, which is consistent with this study’s findings (Fig. 2F). Taken together, the presence of maltol in MBAE may contribute to the extract’s observed ability to prevent or alleviate cisplatin-induced nephrotoxicity.

The assessment of cell viability provides insights into the effectiveness, selectivity, and safety of a drug before examining its molecular mechanisms [29]. In the current study, MBAE showed minimal cytotoxicity in HEK-293 (Fig. 1) cells at all tested concentrations, with the exception of 200 µg/ml, especially at 24 h of treatment. This observation reflects a non-linear, hormetic dose–response. Hormesis is a biphasic dose response characterized by low-dose stimulation and high-dose inhibition, a phenomenon that is highly generalizable across biological models and endpoints [30, 31]. Such U-shaped responses are documented for many phytochemicals, where mild stress at low doses transiently inhibit growth, while higher doses activate adaptive stress responses, including enhanced mitochondrial dehydrogenase activity. Similarly, minimal cytotoxicity was induced in MDA-MB-231 (Fig. 5) cells at the tested extract concentrations, as evidenced by the percentage of viable cells remaining above ~ 80%. Several factors may contribute to this response, the most significant being the polarity-dependent variations in phytoconstituents present in extracts [32]. In a previous study, a more nonpolar acetone extract of *M. balsamina* significantly decreased the viability of MCF-7 breast cancer cells at concentrations ranging from 100 µg/ml to 200 µg/ml [21]. In another study conducted on HT-29 colon cancer cells, the same extract exhibited cytotoxic effects at concentrations ranging from 50 µg/ml to 200 µg/ml [16], which supports the notion that nonpolar extracts are more likely to induce toxicities in cancerous cells than their polar counterparts. The anticancer activities of MBAE have not been explored, perhaps because higher extract concentrations would be required to elicit effects easily achieved by lower concentrations of organic extracts, such as acetone or methanol extracts. The extract’s lack of toxicity in HEK-293 cells highlights its potential biocompatibility with normal cells.

The extract increased the metabolic activity of cisplatin-challenged HEK-293 cells, demonstrated by the enhanced reduction of the WST-8 tetrazolium salt into formazan dye (Fig. 1C and D), which is indicative of increased mitochondrial dehydrogenase activity. This reflects improved mitochondrial function, typically impaired by cisplatin exposure, suggesting that the extract may potentially mitigate cisplatin-induced mitochondrial dysfunction. Furthermore, its ability to enhance mitochondrial dehydrogenase activity in cisplatin-treated cells indicates that the extract may also play a role in reducing oxidative stress, which, in turn, may improve antioxidant defenses to prevent kidney cell apoptosis. In contrast, exposure of MDA-MB-231 breast cancer cells to cisplatin and the extract resulted in a time-dependent decrease in metabolic activity (Fig. 5C and D), evidenced by a reduced conversion of WST-8 tetrazolium salt to formazan dye.

Combination indices (CI) were calculated using the mutually non‑exclusive Chou‑Talalay model, as MBAE and cisplatin are presumed to exert their effects through pharmacologically distinct mechanisms. Cisplatin induces cytotoxicity primarily through DNA crosslinking, activation of the p53 signaling cascade, and mitochondrial apoptosis [33, 34]. In contrast, plant‑derived extracts such as MBAE typically confer cytoprotection through direct free radical scavenging, and inhibition of pro‑apoptotic factors such as Bax and caspase‑3 [35, 36]. The non‑exclusive formula, which accounts for independent or partially independent sites of action, is therefore more appropriate than the mutually exclusive model and provides a more conservative estimate of drug interaction [37, 38]. In HEK‑293 normal kidney cells at 24 h, all combinations produced CI > 1.1 (Table S1), indicating antagonism, which is a pharmacologically desirable outcome for nephroprotection. However, at 48 h (Table S2), CI could not be determined for MBAE concentrations ≥ 400 µg/ml because observed cell viability exceeded 100% of the control, yielding negative fraction affected (Fa) values. The median‑effect equation is derived from the law of mass action and assumes a monotonic dose-response relationship with Fa constrained to the interval (0,1). Negative Fa values, which indicate net cell proliferation rather than inhibition, fall outside this theoretical framework and preclude CI calculation [37]. Some biological factors may account for this observation. In addition to hormesis, MBAE degradation products may accumulate during 48 h of culture under standard cell culture conditions (37 °C, 5% CO₂), potentially generating metabolites with growth‑promoting activity distinct from the parent extract [39]. Such degradation has been reported for various plant‑derived compounds, including phenolic acids and flavonoids, which can undergo oxidation, hydrolysis, or isomerization over extended incubation periods [40]. In the instance of MDA‑MB‑231 breast cancer cells, CI calculation was not possible at either time point, as cisplatin alone at the tested concentration failed to produce a quantifiable cytotoxic effect (viability 98–101%; Fa ≈ 0). The median‑effect equation requires each agent alone to exhibit a measurable effect to calculate the median‑effect dose (Dₘ); when Fa approaches zero, the term Fa/(1‑Fa) approaches zero, rendering Dₘ mathematically undefined [37]. Consequently, higher cisplatin concentrations spanning the full dose‑response continuum are required to determine whether MBAE compromises the antineoplastic activity of cisplatin.

Advanced preclinical 3D cell culture models, such as spheroids, are valuable biomimetics that enable long-term assessment of drug response and serve as excellent tools for bridging the gap between 2D cultures and in vivo models [41]. In the current study, MBAE demonstrated substantial protection against cisplatin toxicity in HEK-293 spheroids, as indicated by increased ATP production (Fig. 1G), measured using the luciferin-luciferase reaction. An effective nephroprotective strategy should also be evaluated under tumor-bearing conditions, as renal protective agents may inadvertently protect tumors by interfering with cisplatin’s anticancer activity [8]. Such a response undermines the suitability of these approaches for mitigating kidney pathology in individuals treated with cisplatin. The findings of this study revealed that extract supplementation preserved the cytotoxic effects of cisplatin in MDA-MB-231 spheroids (Fig. 5G). Evidently, the extract offers long-term nephroprotection without interfering with cisplatin’s anticancer properties, further highlighting its potential as a complementary agent in cisplatin-based chemotherapy. In addition, the extract could improve the safety and efficacy of prolonged cisplatin treatment; however, further investigations in in vivo models under extended cisplatin treatment are imperative to validate these findings.

The paradox of tumorigenesis progression observed after combinatorial treatment with 800 µg/ml for 24 h, simultaneous cell death, and reduced viability is unsurprising. Limited diffusion of oxygen and nutrients ultimately leads to the death of cells within the spheroid core, which is also reflected as decreased cell viability. We can therefore deduce that the extract exerts optimal protective effects and potentiates cisplatin cytotoxicity at its lowest concentration in HEK-293 and MDA-MB-231 spheroids, respectively. This also highlights the potential of the extract further as a modulator of chemotherapy efficacy, warranting further investigation into its mechanistic interactions with cisplatin in tumor microenvironments, both in vitro and in vivo.

Several features differentiate early apoptosis from late apoptosis. These features include cell shrinkage, chromatin condensation, loss of the ΔΨm, and phosphatidylserine (PS) externalization in early apoptosis, while late apoptosis is characterized by DNA fragmentation, membrane blebbing, and loss of plasma membrane integrity [42]. In this study, supplementing cisplatin with the extract increased PS externalization in HEK-293 kidney cells (Fig. 2A and B) synonymous with early apoptosis. In contrast, MDA-MB-231 breast cancer cells (Fig. 6) exhibited loss of membrane integrity as a higher percentage of cells were tagged with Annexin V and propidium iodide (PI), indicative of late apoptosis. Unlike early apoptosis, which is reversible through a process known as anastasis, where the PS is re-internalized upon removal of the apoptotic stimulus, cells in late apoptosis are irreversibly committed to cell death [43]. This data implies that the extract has a level of selective cytotoxicity i.e., it induces reversible apoptosis in HEK-293 cells and irreversible apoptosis in MDA-MB-231 cells, a notion further supported by the reduction of cisplatin toxicity in the HEK-293 2D and 3D spheroid cultures (Fig. 1). The observed divergence between the viability of MDA-MB-231 2D models (Fig. 5) and the incidence of apoptosis (Fig. 6) at 24 h can be attributed to the differences in the assay principles. The CCK 8 assay quantifies mitochondrial metabolic activity (NAD(P)H-dependent dehydrogenase activity), whereas Annexin V and PI are used to identify phosphatidylserine externalization and cellular membrane integrity, which serve as indicators of apoptotic processes. As elucidated in the Assay Guidance Manual [44], an elevation in metabolic activity does not necessarily imply an increase in the cellular population, as cells experiencing stress may enhance mitochondrial function before undergoing apoptotic cell death. Apoptotic cell death is governed by an interplay of proteins, the most studied being Bax and Bcl-2 [45], that regulate the ΔΨm. The extent to which cells are sensitized to apoptotic stimuli depends on the ratios of Bax/Bcl-2 heterodimers or homodimerization [46]. Our findings revealed that the extract supplementation reduced Bax/Bcl-2 ratios in cisplatin-challenged HEK-293 cells (Fig. 2E and F). These findings support the extract`s potential as a complementary agent that decreases the apoptotic machinery that serves as a mode of cisplatin action in kidney pathology.

Cisplatin induces apoptosis in kidney cells by forming DNA crosslinks, eventually disrupting DNA replication and transcription [24]. Upon sensing genotoxic stress, such as DNA damage caused by cisplatin, renal cells exit the G0 phase and enter the cell cycle, where arrest occurs to initiate DNA repair mechanisms [4]. If the damage is not repaired, apoptosis is induced. In this study, the extract reversed cisplatin-induced DNA damage at 24 h (Fig. 3A). A unique cellular response was observed after 48 h of treatment, where an increase in the activation of DNA damage markers H2A.X and pATM was observed (Fig. 3B). Expectedly, this was accompanied by cell cycle halting in the G0/G1-phase, which signals the induction of DDR. Moreover, increasing the supplementation concentration allowed cell cycling. This was shown by a decrease in S-phase and an increase in G2/M phase cell populations (Fig. 4). This was validated by the upregulation of p53 (Fig. 3D) and subsequent p21 protein expression (Fig. 3C), which further upregulated DNA repair mechanisms, including PARP1-regulated base excision repair (Fig. 3C), suggesting that extract supplementation enhances genetic stability in cisplatin-challenged HEK-293 cells. The limitations of this study are that the effects of the extract alone or combination cisplatin were not assessed in normal breast cell models. As a result, it could not be determined whether extract`s cytotoxic effects on MDA-MB-231 were selective or not. In addition, the molecular mechanism induced by extract-only treatment was not investigated. Thus, further studies should be undertaken to determine the combinatory and extract-only treatments on normal breast cells to determine their safety and selectivity. Moreover, the effects of the extract alone on molecular pathways investigated in the study should be assessed to provide an insight on its mechanism of action.

## Conclusions

In conclusion, this study demonstrated that MBAE exhibits significant nephroprotective effects against cisplatin-induced toxicity in HEK-293 cells, both in 2D and 3D models, while preserving or enhancing the anticancer activity of cisplatin in MDA-MB-231 breast cancer cells. Mechanistically, MBAE reduces apoptosis by modulating the Bax/Bcl-2 ratio, enhances DNA repair via the PARP1/p53/p21 axis, and induces cell cycle arrest at G0/G1 and S-phases to facilitate DNA damage repair. These findings position MBAE as a promising complementary agent for cisplatin-based chemotherapy.

## Supplementary Information

Below is the link to the electronic supplementary material.

Supplementary Material 1

## Data Availability

The datasets used and analyzed during the current study are available within the article and its Supplementary Information file **.** Additional raw datasets are available from the corresponding author upon reasonable request.

## Abbreviations

2D: Two-dimensional
3D: Three-dimensional
ATM: Ataxia-telangiectasia mutated
ATR: Ataxia-telangiectasia and Rad3-related protein
Bax: Bcl-2–associated X protein
BCA: Bicinchoninic acid
Bcl-2: B-cell lymphoma 2
CCK-8: Cell Counting Kit-8
DDR: DNA damage response
DRR: DNA repair response
ΔΨm: Mitochondrial transmembrane potential
EDTA: Ethylenediaminetetraacetic acid
FITC: Fluorescein isothiocyanate
GC: Gas chromatography
GC–MS: Gas chromatography–mass spectrometry
HEK-293: Human embryonic kidney 293 cells
HRP: Horseradish peroxidase
MBAE: Momordica balsamina aqueous extract
MS: Mass spectrometry
NCDs: Non-communicable diseases
PARP1: Poly(ADP-ribose) polymerase 1
PBS: Phosphate-buffered saline
PI: Propidium iodide
PVDF: Polyvinylidene difluoride
RIPA: Radioimmunoprecipitation assay buffer
ROS: Reactive oxygen species
SAMRC: South African Medical Research Council
SDS: Sodium dodecyl sulfate
TNBC: Triple Negative Breast Cancer
TP53 / p53: Tumor Tumor protein 53
UV: Ultraviolet
WST-8: Water-soluble tetrazolium salt-8
