# Supplementary material for: Exploring the therapeutic potential of momordica balsamina: A focus on anticancer and nephroprotective effects
Source: Mol Biol Rep. 2026 Jul 20;53(1):1210. doi: 10.1007/s11033-026-12383-9 (PMC13384974; doi:10.1007/s11033-026-12383-9)
Supplement: Supplementary file 1 — Supplementary Material 1 [file 11033_2026_12383_MOESM1_ESM.docx]

**Supplementary Information**

**Contents**

**Supplementary Figure S1. Effect of cisplatin on HEK-293 cell viability**

**Supplementary Table S1. Combination index values following combinatorial treatment for 24 hours**

**Supplementary Table S2. Combination index values following combinatorial treatment for 48 hours**

**Supplementary Figure**

**Supplementary Figure S1**

**
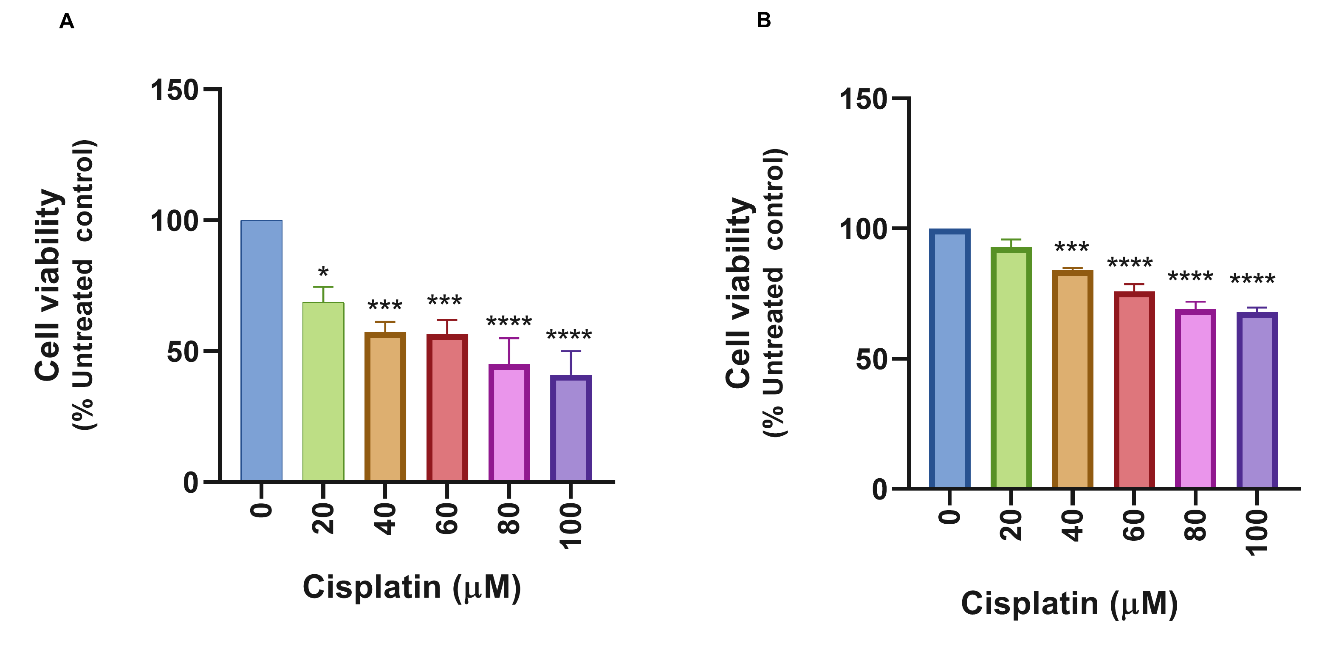
**

**Figure S1. Effect of cisplatin on HEK-293 cell viability at 24 (A) and 48 (B) hours of treatment.** Data are presented as mean ± standard error of the mean (SEM) from three independent experiments performed in duplicate. Data were expressed as the standard error of the mean (SEM) from three independent experiments conducted in duplicates. *p ˂ 0.01 and ***p ≤ 0.0001, ****p < 0.0001 indicate significant differences from untreated control. #p < 0.01, ##p ≤ 0.001, ###p ≤ 0.001, and ####p < 0.0001 denote significant differences relative to cisplatin treated cells.

**Supplementary Tables**

Supplementary Table S1: Combination index values following combinatorial treatment of HEK-293 cells for 24 hours

| **HEK-293 treatment at 24 hours** | | | |
| --- | --- | --- | --- |
| **MBAE (µg/ml)** | **Fa** | **CI** | **Interpretation** |
| 200 | 0.428 | 1.31 | Antagonism |
| 400 | 0.388 | 1.55 | Antagonism |
| 600 | 0.291 | 2.39 | Antagonism |
| 800 | 0.178 | 4.52 | Antagonism |
| 1000 | 0.180 | 4.48 | Antagonism |

Supplementary Table S2: Combination index calculated following combinatorial treatment of HEK-293 cells for 48 hours

| **HEK-293 treatment at 48 hours** | | | |
| --- | --- | --- | --- |
| **MBAE (µg/ml)** | **Fraction affected (Fa)** | **Combination index (CI)** | **Interpretation** |
| 200 | 0.256 | 2.17 | Antagonism |
| 400 | -0.017 | Undefined | No inhibition resulting from Negative Fa |
| 600 | -0.146 | Undefined | No inhibition resulting from Negative Fa |
| 800 | -0.384 | Undefined | No inhibition resulting from Negative Fa |
| 1000 | -0.314 | Undefined | No inhibition resulting from Negative Fa |

CI < 0.9 indicates synergy

CI = 1 indicates additive

CI > 1.1 indicates antagonism
